# Top 100 Most-Cited Studies on MRI and Neuroplasticity in Stroke: A Scoping Review and Bibliometric Analysis of Citation Trends

**DOI:** 10.7759/cureus.91484

**Published:** 2025-09-02

**Authors:** Gabriel Manrique-Gutierrez, Ernesto Roldan-Valadez, Sergey K Ternovoy, Ana G Ramirez-Nava, Aldo Montes-Ugaldo, Jimena Quinzaños-Fresnedo

**Affiliations:** 1 Program of Combined Studies in Medicine (PECEM) Faculty of Medicine, National Autonomous University of Mexico (UNAM), Mexico City, MEX; 2 Division of Research, Instituto Nacional de Rehabilitacion "Luis Guillermo Ibarra Ibarra" (INR), Mexico City, MEX; 3 Department of Radiology, I.M. Sechenov First Moscow State Medical University, Moscow, RUS; 4 Division of Neurological Rehabilitation, Instituto Nacional de Rehabilitación “Luis Guillermo Ibarra Ibarra”, Mexico City, MEX; 5 Division of Neurological Rehabilitation, Instituto Nacional de Rehabilitacion “Luis Guillermo Ibarra Ibarra”, Mexico City, MEX

**Keywords:** bibliometric analysis，visual analysis, cortical reorganization, diffusion tensor imaging (dti), functional magnetic resonance imaging (fmri), human connectomics, intrinsic brain networks, motor recovery, mri magnetic resonance imaging, neuroplasticity, post-stroke recovery

## Abstract

Stroke is a leading cause of long-term disability worldwide. Magnetic resonance imaging (MRI) has become central to understanding neuroplasticity-the brain’s capacity to reorganize after injury-by providing insights into structural and functional changes during recovery. Despite its importance, the intellectual and conceptual foundations of MRI-based neuroplasticity research in stroke remain underexplored.

We conducted a bibliometric analysis of publications indexed in Scopus from January 2004 to December 2024. Eligible studies were original articles and reviews assessing neuroplasticity in stroke using MRI modalities. Citation Classics were defined as ≥100 citations. Performance indicators (citations, citation rate per year, active years, authorship, article type) were compared using Mann-Whitney U and Chi-square tests. Science mapping was performed with Biblioshiny (K-Synth Srl, Naples, Italy) and VOSviewer (Centre for Science and Technology Studies, Leiden University, Leiden, The Netherlands) to identify intellectual, conceptual, and collaborative structures.

From 854 initial records, 100 most-cited articles were included (59-1,274 citations). Citation Classics (n = 62) had significantly higher mean citation counts and rates per year than non-Classics (p < 0.0001). Reviews predominated among Classics, while original research was more frequent in non-Classics. High-impact journals (*Brain*, *Stroke*, *Neurorehabilitation and Neural Repair*, and *NeuroImage*) accounted for the most influential articles, with strong correlations between journal metrics and citation performance. Leading institutions were Harvard Medical School, University of Cambridge, and University of California, Irvine, while the United States, Germany, and the United Kingdom dominated global contributions. Emerging countries, including China and Japan, showed increasing participation. Science mapping revealed clusters on motor recovery, cortical reorganization, language rehabilitation, and emerging areas such as connectomics and machine learning.

This study provides a comprehensive overview of MRI-based neuroplasticity research in stroke, identifying seminal works, leading contributors, and evolving research themes. While citation activity has declined in recent years, advances in connectomics, multi-modal imaging, and computational approaches offer promising directions. Expanding global collaborations and addressing underrepresented regions will be critical for inclusive progress. MRI remains central to bridging mechanistic insights with clinical applications, guiding innovation in personalized stroke rehabilitation.

## Introduction and background

Stroke is an acute vascular condition that produces neurological deficits [1]. Traditionally, it was considered a localized disease affecting only the infarcted region [2]. Advances in neuroanatomy and physiology, however, have shown that focal lesions disrupt activity in distant areas, including the non-affected hemisphere, through a phenomenon known as connectional diaschisis [3]. Stroke remains one of the leading causes of long-term disability worldwide, imposing a substantial burden on patients, caregivers, and healthcare systems [4-6].

Recovery after stroke is mediated by multiple factors. Spontaneous recovery predominates during the first three to six months, while rehabilitation is essential in both acute and chronic stages to consolidate functional gains [7-9]. A critical substrate for these processes is neuroplasticity, defined as the brain’s ability to reorganize and form new connections after injury. Neuroplasticity reflects the nervous system’s intrinsic capacity to adapt to environmental changes, experiences, and external stimuli, enabling compensatory mechanisms in the presence of structural damage [7,8,10,11]. After a stroke, several biological processes, including neurogenesis, synaptogenesis, neuronal reorganization, and compensatory pathways, contribute to recovery [12-15].

To study these mechanisms, a range of non-invasive techniques has been used. Electroencephalography (EEG), magnetoencephalography (MEG), positron emission tomography (PET), transcranial magnetic stimulation (TMS), and transcranial direct current stimulation (tDCS) are widely applied to assess brain activity, metabolism, and cortical excitability [2,8,16]. Among these, magnetic resonance imaging (MRI) has emerged as a cornerstone tool for evaluating neuroplasticity.

MRI provides high-resolution structural and functional information, making it uniquely suited to investigate neural reorganization after stroke. Submodalities such as diffusion-weighted imaging (DWI) and diffusion tensor imaging (DTI) allow visualization of white matter tracts [17], assessment of fiber orientation, and mapping of structural connectivity. Functional MRI (fMRI) measures hemodynamic responses to neural activity, enabling both task-based and resting-state network mapping [18]. Magnetic resonance spectroscopy (MRS) provides metabolic insights by quantifying biochemical changes associated with cerebral ischemia [2,10,16,19]. Together, these techniques establish MRI as a versatile platform for exploring brain plasticity.

Bibliometric analysis, defined as the quantitative study of publications and citations to identify influential research and emerging trends, offers a systematic way to assess the intellectual development of this field. Unlike traditional literature reviews, bibliometric studies enable both performance analysis, focusing on output and citation metrics, and science mapping, which identifies conceptual linkages and collaborative networks [20-22].

In this study, we applied a bibliometric approach to evaluate two decades (2004-2024) of MRI-based research on neuroplasticity in stroke. By integrating performance analysis with science mapping, we aimed to capture not only publication and citation trends but also the intellectual, conceptual, and collaborative structures shaping this area. This scoping approach provides a comprehensive overview of how MRI has informed understanding of post-stroke neuroplasticity, offering neurologists, rehabilitation specialists, and imaging researchers curated access to seminal contributions, thematic clusters, and future research priorities.

## Review

Methods

Search Methodology and Data Sources

A comprehensive literature search was conducted in Scopus (https://www.scopus.com/home.uri) on October 18, 2024. The timeframe was restricted to publications between January 2004 and December 2024. Impact Factor (IF) values and quartile classifications (Q1-Q4) were retrieved from Clarivate Analytics’ Journal Citation Reports (https://jcr.clarivate.com/jcr). The search strategy was organized into four domains: MRI modalities, neuroplasticity, stroke, and exclusions (Table 1).

**Table 1:** Comprehensive Scopus Search Strategy for MRI-Based Neuroplasticity Research in Stroke. The search algorithm was structured into four domains (MRI modalities, neuroplasticity, stroke, and exclusions), with grouped keywords and Boolean operators tailored to capture the breadth of the field. This approach ensured inclusion of diverse MRI submodalities (e.g., fMRI, DTI, MRS) and their applications in neuroplasticity assessments among stroke patients, while systematically excluding irrelevant or non-human studies. The detailed strategy enhances transparency and replicability of the bibliometric analysis. fMRI: functional MRI; DTI: diffusion tensor imaging; MRS: magnetic resonance spectroscopy

Primary Focus and Context	Explanation	Scopus Search Algorithm	Boolean Connector
MRI Techniques and Modalities	Keywords related to MRI neuroimaging modalities used to assess neuroplasticity in stroke patients.	(“MRI” OR “Magnetic Resonance Imaging” OR “Structural MRI” OR “Structural Magnetic Resonance Imaging” OR “dMRI” OR “Diffusion Magnetic Resonance Imaging” OR “DW-MRI” OR “Diffusion-Weighted Magnetic Resonance Imaging” OR “DWI” OR “Diffusion-Weighted Imaging” OR “DTI” OR “Diffusion Tensor Imaging” OR “DTI Tractography” OR “fMRI” OR “Functional Magnetic Resonance Imaging” OR “t-fMRI” OR “tfMRI” OR “Task Based fMRI” OR “rs-fMRI” OR “rsfMRI” OR “Resting State fMRI” OR “MRS” OR “Magnetic Resonance Spectroscopy”)	AND
Application (Neuroplasticity)	Keywords related to the assessment of neuroplasticity in stroke.	(“Neuroplasticity” OR “Neuronal Plasticity” OR “Neural Plasticity” OR “Brain Plasticity” OR “Brain Reorganization” OR “Brain Restructuring”)	AND
Target Area (Stroke)	Keywords related to stroke and cerebrovascular accident.	(“Stroke” OR “Ischemic Stroke” OR “Brain Ischemia” OR “Brain Infarct” OR “Hemorrhagic Stroke” OR “Brain Hemorrhage” OR “Brain Vascular Accident”)	AND
Exclusion Criteria	Keywords to exclude studies unrelated to neuroplasticity in stroke.	(“Traumatic Brain Injury” OR “Acquired Brain Injury” OR “Concussion” OR “Anoxic Ischemic Encephalopathy” OR “Spinal Cord Injury” OR “Chronic Pain” OR “Neurodegenerative Disorders” OR “Dementia” OR “Mild Cognitive Impairment” OR “Alzheimer” OR “Perinatal Stroke” OR “Cerebral Palsy” OR “Depression” OR “Schizophrenia”)	AND NOT
Complete Scopus Search Algorithm			
( TITLE-ABS-KEY ( ( "MRI" OR "Magnetic Resonance Imaging" OR "Structural MRI" OR "Structural Magnetic Resonance Imaging" OR "dMRI" OR "Diffusion Magnetic Resonance Imaging" OR "DW-MRI" OR "Diffusion-Weighted Magentic Resonance Imaging" OR "DWI" OR "Diffusion Weighted Imaging" OR "DTI" OR "Diffusion Tensor Imaging" OR "DTI Tractography" OR "fMRI" OR "Functional Magnetic Resonance Imaging" OR "t-fMRI" OR "tfMRI" OR "Task Based fMRI" OR "rs-fMRI" OR "rsfMRI" OR "Resting State fMRI" OR "MRS" OR "Magnetic Resonance Spectroscopy" ) ) AND TITLE-ABS-KEY ( ( "Neuroplasticity" OR "Neuronal Plasticity" OR "Neural Plasticity" OR "Brain Plasticity" OR "Brain Reorganization" OR "Brain Restructuring" ) ) AND TITLE-ABS-KEY ( ( "Stroke" OR "Ischemic Stroke" OR "Brain Ischemia" OR "Brain Infarct" OR "Hemorrhagic Stroke" OR "Brain Hemorrhage" OR "Brain Vascular Accident" ) ) AND NOT TITLE-ABS-KEY ( ( "Traumatic Brain Injury" OR "Acquired Brain Injury" OR "Concussion" OR "Anoxic Ischemic Encephalopathy" OR "Spinal Cord Injury" OR "Chronic Pain" OR "Neurodegenerative Disorders" OR "Dementia" OR "Mild Cognitive Impairment" OR "Alzheimer" OR "Perinatal Stroke" OR "Cerebral Palsy" OR "Depression" OR "Schizophrenia" ) ) ) AND PUBYEAR > 2003 AND PUBYEAR			

Inclusion and Exclusion Criteria

Eligible studies were original research articles or reviews evaluating neuroplasticity in stroke survivors using MRI modalities. Exclusion criteria were: studies involving animal models or conditions other than stroke; non-peer-reviewed outputs (e.g., conference proceedings, editorials, short surveys, letters, book chapters); and publications not written in English.

Screening and Selection Process

**Figure 1:**
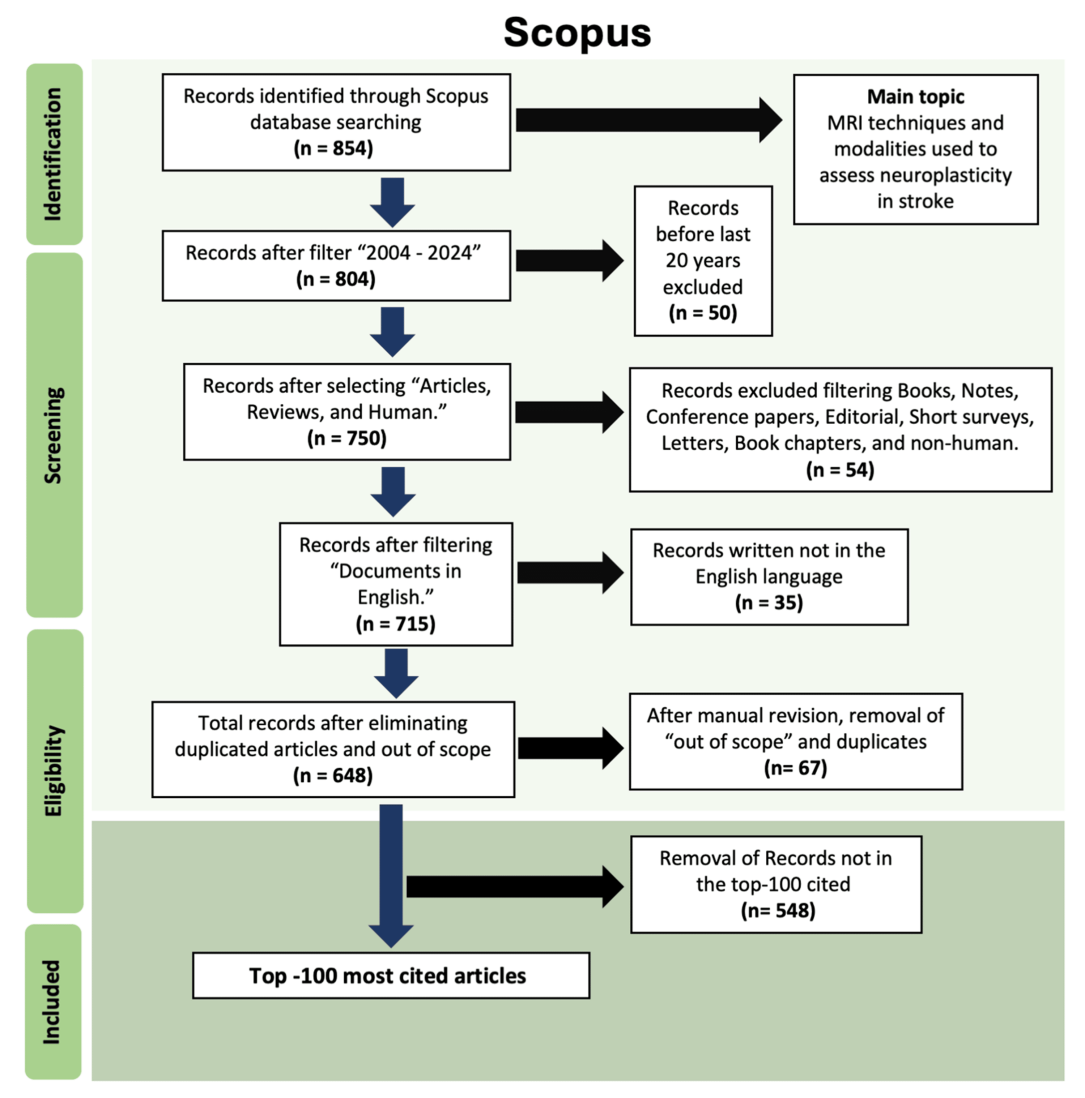
Workflow of the Bibliometric Analysis. Stepwise methodology for identifying the top 100 most-cited articles on MRI-based neuroplasticity in stroke. The PRISMA-style diagram illustrates database retrieval (854 records), application of inclusion/exclusion criteria, and final selection for analysis.

The search initially identified 854 records. After removing duplicates, titles and abstracts were screened independently by two reviewers (G.M.G. and E.R.V.) to assess compliance with eligibility criteria. Disagreements were resolved by consensus. Following full-text review, the 100 most-cited articles were retained for analysis. This bibliometric study was not registered in PROSPERO, as it does not synthesize clinical outcomes, but it followed the PRISMA-ScR (Scoping Review) guidelines [20] to ensure methodological transparency and replicability, the selection pathway is illustrated in Figure 1 (PRISMA flow diagram).

Definition of Bibliometric Analysis

Bibliometric analysis (BA) is a quantitative approach to evaluating authorship patterns, journal impact, and publication influence within a research field [21,22]. By analyzing citation data, BA identifies seminal studies, research trends, institutions, and collaborative networks.

Identification of Citation Classics

Following Garfield’s original definition [23], articles with ≥100 citations were classified as Citation Classics. Articles with <100 citations were categorized as Non-Classics.

Bibliometric Analysis Techniques

Two complementary approaches were applied [21]: (1) Performance analysis quantified research output through the following indicators: total publications (TP), number of active years of publication (NAY), articles per active year (APY), number of contributing authors (NCA), and classification as sole-authored (SA) or co-authored (CA). (2) Science mapping explored intellectual structures, conceptual linkages, and collaborative networks through co-citation, keyword co-occurrence, and co-authorship analysis.

Statistical Analysis

Comparisons between Citation Classics and non-Classics were performed using: Chi-squared test (χ²) for categorical variables; and Mann-Whitney U test for nonparametric continuous variables.

For each group, means, standard deviations (SD), and 95% confidence intervals (CIs) were calculated. Journal bibliometric metrics (Impact Factor (IF), CiteScore, SCImago Journal Rank (SJR), and Source Normalized Impact per Paper (SNIP)) were summarized with medians, quartiles, and interquartile ranges (IQRs). Associations between bibliometric measures and citation performance were assessed with Spearman’s ρ, and scatterplots were generated for visualization. Statistical significance was set at p < 0.05 [24,25].

Software: Data handling was performed with Microsoft Excel v.16.67 (Microsoft Corporation, Redmond, WA, USA). Statistical analyses were conducted in R v.4.4.1 (The R Foundation, Vienna, Austria). The Biblioshiny package (K-Synth Srl, Naples, Italy) [26] was used for performance analysis. VOSviewer v.1.6.20 (Centre for Science and Technology Studies, Leiden University, Leiden, The Netherlands) [27] generated science mapping visualizations. Thresholds were defined as follows: ≥10 citations per author, ≥2 documents for institutions, countries, or journals, and ≥5 keyword co-occurrences.

Results

Performance Analysis

Retrieval and Selection of Articles:* *The Scopus search yielded 854 records related to MRI-based neuroplasticity research in stroke. Restricting the timeframe to 2004-2024 reduced this to 804 records. Exclusion of conference papers, books, editorials, short surveys, letters, and non-human studies left 750 records. Limiting to English-language publications yielded 715 records. After duplicate removal and manual screening, 648 articles remained. From these, the 100 most-cited articles were retained for analysis (Table 2). The selection workflow is depicted in Figure 1 (PRISMA flow diagram).

**Table 2:** Top 100 Most-Cited Articles on MRI-Based Neuroplasticity Research in Stroke. This table presents the 100 most-cited articles published between 2004 and 2024 that used magnetic resonance imaging (MRI) to evaluate neuroplasticity in stroke. Articles are ranked by citation count and include key bibliometric details (authors, year of publication, journal, and total citations). These studies represent seminal contributions that have shaped the intellectual and clinical understanding of stroke recovery and brain reorganization. BCI: brain-computer interface; fMRI: functional MRI; rTMS: repetitive TMS; tDCS: transcranial direct current stimulation; TMS: transcranial magnetic stimulation; VR: virtual reality

Ranking	Title	1st Author	Journal	Publication Year	Times Cited	Average Citation per Year
1	The plastic human brain cortex [8]	Pascual-Leone A.	Annual Review of Neuroscience	2005	1274	64
2	Dynamics of language reorganization after stroke [28]	Saur D.	Brain	2006	876	46
3	Cognitive motor processes: The role of motor imagery in the study of motor representations [29]	Munzert J.	Brain Research Reviews	2009	587	37
4	Motor imagery: A backdoor to the motor system after stroke? [30]	Sharma N.	Stroke	2006	583	31
5	Dynamic functional reorganization of the motor execution network after stroke [14]	Wang L.	Brain	2010	527	35
6	Robot-based hand motor therapy after stroke [31]	Takahashi C.D.	Brain	2008	518	30
7	Reorganization of the human ipsilesional premotor cortex after stroke [32]	Fridman E.A.	Brain	2004	385	18
8	Motor system activation after subcortical stroke depends on corticospinal system integrity [33]	Ward N.S.	Brain	2006	357	19
9	Virtual reality-induced cortical reorganization and associated locomotor recovery in chronic stroke: An experimenter-blind randomized study [34]	You S.H.	Stroke	2005	351	18
10	Renewal of the neurophysiology of language: Functional neuroimaging [35]	Démonet J.-F.	Physiological Reviews	2005	347	17
11	Remodeling the brain: plastic structural brain changes produced by different motor therapies after stroke [36]	Gauthier L.V.	Stroke; a journal of cerebral circulation	2008	328	19
12	Motor rehabilitation and brain plasticity after hemiparetic stroke [37]	Schaechter J.D.	Progress in Neurobiology	2004	325	15
13	Motor recovery and cortical reorganization after mirror therapy in chronic stroke patients: A phase II randomized controlled trial [38]	Michielsen M.E.	Neurorehabilitation and Neural Repair	2011	297	21
14	Ankle dorsiflexion as an fMRI paradigm to assay motor control for walking during rehabilitation [39]	Dobkin B.H.	NeuroImage	2004	258	12
15	Functionally Specific Reorganization in Human Premotor Cortex [40]	O'Shea J.	Neuron	2007	255	14
16	Sleep-dependent motor memory plasticity in the human brain [41]	Walker M.P.	Neuroscience	2005	237	12
17	Treadmill exercise activates subcortical neural networks and improves walking after stroke: A randomized controlled trial [42]	Luft A.R.	Stroke	2008	229	13
18	Motor imagery after stroke: Relating outcome to motor network connectivity [43]	Sharma N.	Annals of Neurology	2009	224	14
19	Are networks for residual language function and recovery consistent across aphasic patients? [44]	Turkeltaub P.E.	Neurology	2011	223	16
20	Resting state changes in functional connectivity correlate with movement recovery for BCI and robot-Assisted upper-extremity training after stroke [45]	Várkuti B.	Neurorehabilitation and Neural Repair	2013	217	18
21	Non-invasive mapping of corticofugal fibres from multiple motor areas - Relevance to stroke recovery [46]	Newton J.M.	Brain	2006	214	11
22	Recovery from stroke: Current concepts and future perspectives [7]	Grefkes C.	Neurological Research and Practice	2020	209	42
23	Motor cortex stimulation for the enhancement of recovery from stroke: A prospective, multicenter safety study [47]	Brown J.A.	Neurosurgery	2006	197	10
24	Extent of bilateral neuronal network reorganization and functional recovery in relation to stroke severity [48]	van Meer M.P.A.	Journal of Neuroscience	2012	188	14
25	Efficient neuroplasticity induction in chronic stroke patients by an associative brain-computer interface [49]	Mrachacz-Kersting N.	Journal of Neurophysiology	2016	184	20
26	Combination of brain-computer interface training and goal-directed physical therapy in chronic stroke: A case report [50]	Broetz D.	Neurorehabilitation and Neural Repair	2010	183	12
27	Cortical plasticity following motor skill learning during mental practice in stroke [51]	Page S.J.	Neurorehabilitation and Neural Repair	2009	164	10
28	Training and exercise to drive poststroke recovery [52]	Dobkin B.H.	Nature Clinical Practice Neurology	2008	158	9
29	Chronic stroke recovery after combined BCI training and physiotherapy: A case report [53]	Caria A.	Psychophysiology	2011	156	11
30	Movement-dependent stroke recovery: A systematic review and meta-analysis of TMS and fMRI evidence [54]	Richards L.G.	Neuropsychologia	2008	156	9
31	Left hemisphere plasticity and aphasia recovery [55]	Fridriksson J.	NeuroImage	2012	156	12
32	Neuroplasticity of language networks in aphasia: Advances, updates, and future challenges [56]	Kiran S.	Frontiers in Neurology	2019	156	26
33	Adult Visual Cortical Plasticity [57]	Gilbert C.	Neuron	2012	153	12
34	Functional MRI follow-up study of language processes in healthy subjects and during recovery in a case of aphasia [58]	Fernandez B.	Stroke	2004	152	7
35	Functional reorganization of the cerebral motor system after stroke [59]	Ward N.S.	Current Opinion in Neurology	2004	150	7
36	Bilateral and unilateral arm training improve motor function through differing neuroplastic mechanisms: A single-blinded randomized controlled trial [60]	Whitall J.	Neurorehabilitation and Neural Repair	2011	150	11
37	Neuroplasticity and brain repair after stroke [61]	Cramer S.C.	Current Opinion in Neurology	2008	150	9
38	Structural and functional plasticity in the somatosensory cortex of chronic stroke patients [62]	Schaechter J.D.	Brain	2006	147	8
39	The right hemisphere is not unitary in its role in aphasia recovery [63]	Turkeltaub P.E.	Cortex	2012	142	11
40	Integrated technology for evaluation of brain function and neural plasticity [64]	Rossini P.M.	Physical Medicine and Rehabilitation Clinics of North America	2004	138	7
41	Two different reorganization patterns after rehabilitative therapy: An exploratory study with fMRI and TMS [65]	Hamzei F.	NeuroImage	2006	138	7
42	Predicting behavioural response to TDCS in chronic motor stroke [66]	O'Shea J.	NeuroImage	2014	137	12
43	Structural changes induced by daily music listening in the recovering brain after middle cerebral artery stroke: A voxel-based morphometry study [67]	Särkämö T.	Frontiers in Human Neuroscience	2014	135	12
44	Brain networks and their relevance for stroke rehabilitation [2]	Guggisberg A.G.	Clinical Neurophysiology	2019	130	22
45	Impact of alertness training on spatial neglect: A behavioural and fMRI study [68]	Thimm M.	Neuropsychologia	2006	126	7
46	Stem cell therapy: A clinical trial of stroke [69]	Bhasin A.	Clinical Neurology and Neurosurgery	2013	124	10
47	Functional plasticity induced by mirror training: The mirror as the element connecting both hands to one hemisphere [70]	Hamzei F.	Neurorehabilitation and Neural Repair	2012	122	9
48	Leap Motion-based virtual reality training for improving motor functional recovery of upper limbs and neural reorganization in subacute stroke patients [71]	Wang Z.-R.	Neural Regeneration Research	2017	119	15
49	Comparison of finger tracking versus simple movement training via telerehabilitation to alter hand function and cortical reorganization after stroke [72]	Carey J.R.	Neurorehabilitation and Neural Repair	2007	118	7
50	Activity in preserved left hemisphere regions predicts anomia severity in aphasia [73]	Fridriksson J.	Cerebral Cortex	2010	117	8
51	Efficacy and brain imaging correlates of an immersive motor imagery BCI-driven VR system for upper limb motor rehabilitation: A clinical case report [74]	Vourvopoulos A.	Frontiers in Human Neuroscience	2019	117	20
52	Effects of high- and low-frequency repetitive transcranial magnetic stimulation on motor recovery in early stroke patients: Evidence from a randomized controlled trial with clinical, neurophysiological and functional imaging assessments [75]	Du J.	NeuroImage: Clinical	2019	116	19
53	Induction of neuroplasticity and recovery in post-stroke aphasia by non-invasive brain stimulation [76]	Shah P.P.	Frontiers in Human Neuroscience	2013	113	9
54	Neuroimaging experimental studies on brain plasticity in recovery from stroke [77]	Rossini P.M.	Europa Medicophysica	2007	111	6
55	Autologous Mesenchymal Stem Cells Improve Motor Recovery in Subacute Ischemic Stroke: a Randomized Clinical Trial [78]	Jaillard A.	Translational Stroke Research	2020	110	22
56	Functional connectivity in relation to motor performance and recovery after stroke [79]	Westlake K.P.	Frontiers in Systems Neuroscience	2011	108	8
57	Enduring representational plasticity after somatosensory stimulation [80]	Wu C.W.-H.	NeuroImage	2005	107	5
58	Shaping Early Reorganization of Neural Networks Promotes Motor Function after Stroke [81]	Volz L.J.	Cerebral Cortex	2016	107	12
59	Simvastatin and atorvastatin improve neurological outcome after experimental intracerebral hemorrhage [82]	Karki K.	Stroke	2009	105	7
60	Neuroplasticity and constraint-induced movement therapy [83]	Mark V.W.	Europa Medicophysica	2006	102	5
61	Enhanced cortical activation in the contralesional hemisphere of chronic stroke patients in response to motor skill challenge [84]	Schaechter J.D.	Cerebral Cortex	2008	101	6
62	A macroscopic view of microstructure: Using diffusion-weighted images to infer damage, repair, and plasticity of white matter [85]	Concha L.	Neuroscience	2014	97	9
63	Motor cortex excitability and connectivity in chronic stroke: a multimodal model of functional reorganization [86]	Volz L.J.	Brain Structure and Function	2015	96	10
64	Neuroplastic changes following rehabilitative training correlate with regional electrical field induced with tDCS [87]	Halko M.A.	NeuroImage	2011	95	7
65	Diffusion tensor imaging and fiber tractography in acute stroke [88]	Mukherjee P.	Neuroimaging Clinics of North America	2005	94	5
66	A review of transcranial magnetic stimulation and multimodal neuroimaging to characterize post-stroke neuroplasticity [89]	Auriat A.M.	Frontiers in Neurology	2015	94	9
67	Neural plasticity and treatment-induced recovery of sentence processing in agrammatism [90]	Thompson . C.K.	Neuropsychologia	2010	92	6
68	Functional MRI correlates of lower limb function in stroke victims with gait impairment [91]	Enzinger C.	Stroke	2008	92	5
69	Neural recruitment associated with anomia treatment in aphasia [92]	Fridriksson J.	NeuroImage	2006	91	5
70	Enhanced Interhemispheric Functional Connectivity Compensates for Anatomical Connection Damages in Subcortical Stroke [93]	Liu J.	Stroke	2015	91	9
71	Neuroimaging and recovery of language in aphasia [94]	Thompson C.K.	Current Neurology and Neuroscience Reports	2008	87	5
72	Music supported therapy promotes motor plasticity in individuals with chronic stroke [95]	Ripollés P.	Brain Imaging and Behavior	2016	85	9
73	Functional MRI and Diffusion Tensor Imaging of Brain Reorganization After Experimental Stroke [96]	Dijkhuizen R.M.	Translational Stroke Research	2012	83	6
74	Post-lesional cerebral reorganisation: Evidence from functional neuroimaging and transcranial magnetic stimulation [97]	Bütefisch C.M.	Journal of Physiology Paris	2006	80	4
75	Lesion-induced and training-induced brain reorganization [98]	Liepert J.	Restorative Neurology and Neuroscience	2004	79	4
76	Cortical reorganization - Effects of intensive therapy: Results from prospective functional imaging studies [99]	Nelles G.	Restorative Neurology and Neuroscience	2004	78	4
77	Evolution of fMRI activation in the perilesional primary motor cortex and cerebellum with rehabilitation training-related motor gains after stroke: A pilot study [100]	Dong Y.	Neurorehabilitation and Neural Repair	2007	77	4
78	Structural damage and functional reorganization in Ipsilesional M1 in well-recovered patients with subcortical stroke [101]	Zhang J.	Stroke	2014	76	7
79	Therapy-induced neuroplasticity in chronic aphasia [102]	Marcotte K.	Neuropsychologia	2012	76	6
80	Human adult cortical reorganization and consequent visual distortion [103]	Dilks D.D.	Journal of Neuroscience	2007	76	4
81	Neural correlates of proprioceptive integration in the contralesional hemisphere of very impaired patients shortly after a subcortical stroke: An fMRI study [104]	Dechaumont-Palacin S.	Neurorehabilitation and Neural Repair	2008	75	4
82	Structural plasticity in adulthood with motor learning and stroke rehabilitation [105]	Sampaio-Baptista C.	Annual Review of Neuroscience	2018	75	11
83	Mechanism of Kinect-based virtual reality training for motor functional recovery of upper limbs after subacute stroke [106]	Bao X.	Neural Regeneration Research	2013	74	6
84	Changes in white matter integrity follow excitatory rTMS treatment of post-stroke aphasia [107]	Allendorfer J.B.	Restorative Neurology and Neuroscience	2012	70	5
85	Reorganization of syntactic processing following left-hemisphere brain damage: Does right-hemisphere activity preserve function? [108]	Tyler L.K.	Brain	2010	70	5
86	Functional magnetic resonance imaging study on dysphagia after unilateral hemispheric stroke: A preliminary study [109]	Li S.	Journal of Neurology, Neurosurgery and Psychiatry	2009	68	4
87	Acute ischaemic stroke alters the brain's preference for distinct dynamic connectivity states [110]	Bonkhoff A.K.	Brain	2020	68	14
88	Functional neuroimaging in stroke recovery and neurorehabilitation: Conceptual issues and perspectives [111]	Carey L.M.	International Journal of Stroke	2007	66	4
89	Cortical Reorganization Following Modified Constraint-Induced Movement Therapy: A Study of 4 Patients With Chronic Stroke [112]	Szaflarski J.P.	Archives of Physical Medicine and Rehabilitation	2006	66	3
90	A paradox: After stroke, the non-lesioned lower limb motor cortex may be maladaptive [113]	Madhavan S.	European Journal of Neuroscience	2010	66	4
91	Activity in the peri-infarct rim in relation to recovery from stroke [114]	Cramer S.C.	Stroke	2006	65	3
92	A review of diffusion tensor imaging studies on motor recovery mechanisms in stroke patients [115]	Jang S.H.	NeuroRehabilitation	2011	62	4
93	When right is all that is left: Plasticity of right-hemisphere tracts in a young aphasic patient [116]	Zipse L.	Annals of the New York Academy of Sciences	2012	62	5
94	fMRI analysis of ankle movement tracking training in subject with stroke [117]	Carey J.R.	Experimental Brain Research	2004	61	3
95	Effects of Virtual Reality Intervention on Neural Plasticity in Stroke Rehabilitation: A Systematic Review [118]	Hao J.	Archives of Physical Medicine and Rehabilitation	2022	60	20
96	The neural correlates of semantic feature analysis in chronic aphasia: Discordant patterns according to the etiology [119]	Marcotte K.	Seminars in Speech and Language	2010	60	4
97	Effects of home-based telerehabilitation in patients with stroke: A randomized controlled trial [120]	Chen J.	Neurology	2020	59	12
98	Functional MRI of swallowing: From neurophysiology to neuroplasticity [121]	Malandraki G.A.	Head and Neck	2011	59	4
99	Neuroplasticity in Post-Stroke Aphasia: A Systematic Review and Meta-Analysis of Functional Imaging Studies of Reorganization of Language Processing [122]	Wilson S.M.	Neurobiology of Language	2020	59	12
100	Brain activity associated with stimulation therapy of the visual borderzone in hemianopic stroke patients [123]	Marshall R.S.	Neurorehabilitation and Neural Repair	2008	57	6

**Figure 2:**
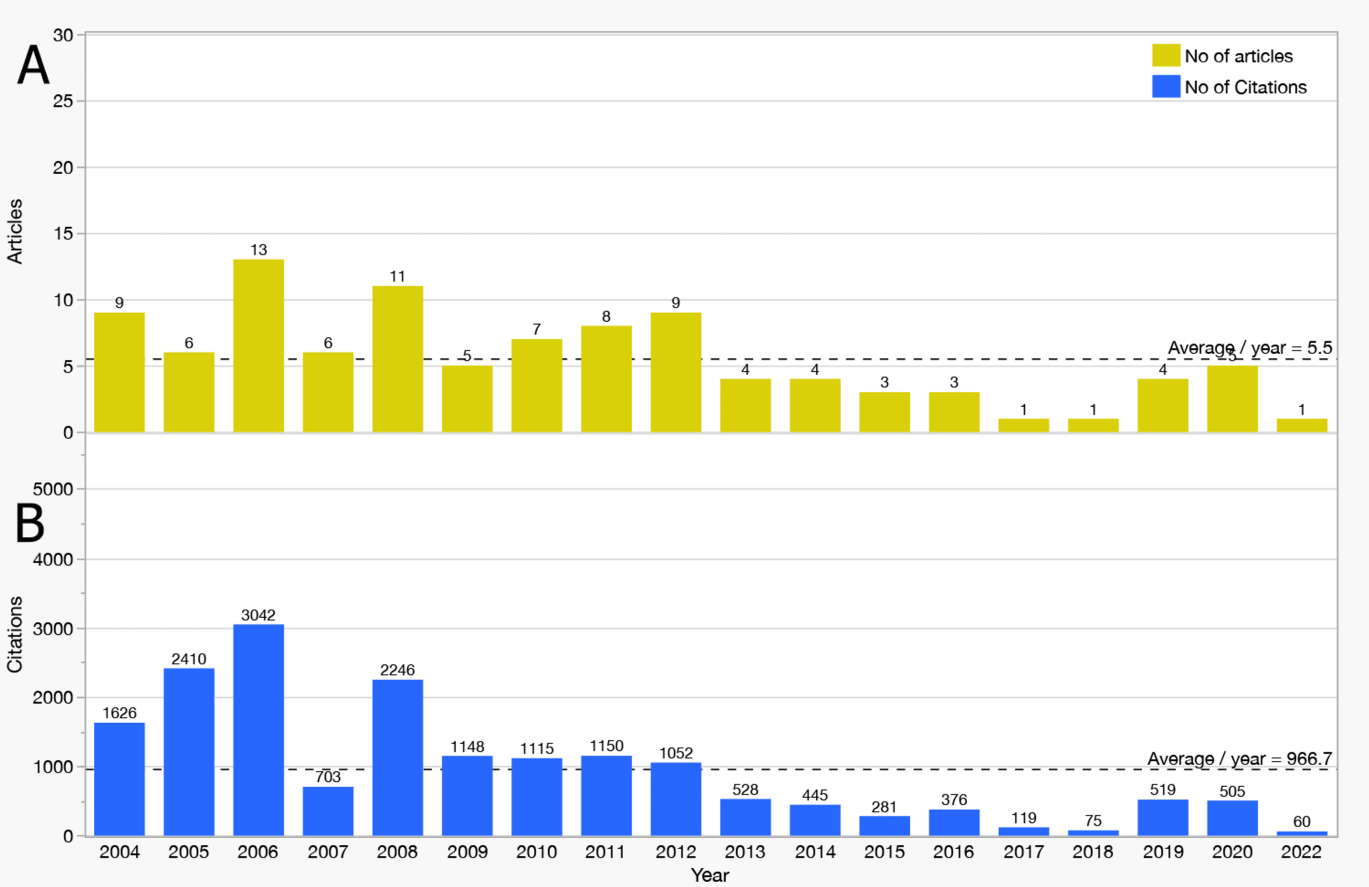
Yearly Trends in Publications and Citations (2004–2024). (A) Annual publication counts, peaking in 2006 with 13 articles, followed by a gradual decline. (B) Annual citation counts, peaking in 2006 with 3,042 citations, reflecting the enduring impact of seminal studies.

Yearly Publications and Citations: Annual publication output showed fluctuations, with a peak of 13 articles in 2006 (Figure 2A). After 2012, output declined, stabilizing at fewer than five publications per year after 2014. The overall mean publication rate across the two decades was 5.5 articles per year.

Citation activity followed a similar trajectory, peaking at 3,042 citations in 2006 (Figure 2B). Despite subsequent declines, modest rebounds in 2019 and 2020 reflected the enduring influence of foundational works. On average, the dataset accumulated 967 citations per year, underscoring the sustained relevance of seminal MRI studies in stroke neuroplasticity.

Citation Classics vs. Non-Classics: Of the 100 analyzed articles, 62 qualified as Citation Classics (≥100 citations) and 38 as Non-Classics (<100 citations). Citation Classics accrued a higher mean citation count (234.1 ± 198.4; 95% CI, 184.9-283.3) compared with Non-Classics (75.9 ± 12.4; 95% CI, 71.8-80.0; p < 0.0001). Similarly, the average citation rate per year was significantly higher among Classics (15.7 ± 10.9; 95% CI, 13.0-18.4) than Non-Classics (6.4 ± 3.6; 95% CI, 5.3-7.5; p < 0.0001) (Table 3).

**Table 3:** Comparison of Citation Classics and Non-classics Based on Continuous Performance Metrics. This table compares continuous performance variables (citations, citations per year, years active, and number of authors) between Citation Classics (≥100 citations) and Non-classics (<100 citations). Statistical comparisons were performed with the Mann–Whitney U test. Values for “All articles” are descriptive summaries of the entire dataset and were not included in statistical testing. A p-value < 0.05 was considered statistically significant.

Performance variables	All articles Mean (SD) [95% CI]	Citation Classic (N = 62) Mean (SD) [95% CI]	Non-classic (N = 38) Mean (SD) [95% CI]	p-value
Citations	174.0 (174.0) [140.1–207.9]	234.1 (198.4) [184.0–284.2]	75.9 (12.4) [71.8–80.0]	< 0.0001*
Average citation per year	12.2 (9.9) [10.2–14.2]	15.7 (10.9) [13.0–18.4]	6.4 (3.6) [5.2–7.6]	< 0.0001*
Years active	14.8 (4.7) [13.8–15.8]	15.3 (4.6) [14.1–16.5]	14.0 (4.9) [12.4–15.6]	0.162
Number of authors	5.9 (5.1) [4.9–6.9]	6.3 (5.9) [4.8–7.8]	5.3 (3.4) [4.2–6.4]	0.295

No significant difference was found in years active (15.3 ± 4.6 vs. 14.0 ± 4.9; p = 0.162) or number of authors per article (6.3 ± 5.9 vs. 5.3 ± 3.4; p = 0.295).

Authorship patterns revealed that 93% of all publications were co-authored, with no significant difference between groups (p = 0.279). In terms of article type, original research comprised 73% of the dataset and reviews 27%, with no significant variation between groups (p = 0.904) (Table 4).

**Table 4:** Comparison of Citation Classics and Non-classics Based on Categorical Performance Variables. This table compares categorical variables (type of authorship and article type) between Citation Classics (≥100 citations) and Non-classics (<100 citations). Statistical comparison performed with the Chi-squared test (χ² test). A p-value < 0.05 was considered statistically significant. Values for “All articles” are descriptive summaries of the entire dataset and were not included in statistical testing.

Performance variables	All articles N (%) [95% CI]	Citation Classic N (%) [95% CI]	Non-classic N (%) [95% CI]	p-value
Authorship				
Co-authored publications	93 (93.0%) [86.1–97.1]	59 (63.4%) [50.9–74.7]	34 (36.6%) [25.3–49.1]	0.279
Sole-authored publications	7 (7.0%) [2.9–13.9]	3 (42.9%) [9.9–81.6]	4 (57.1%) [18.4–90.1]	
Type of article				
Original	73 (73.0%) [63.3–81.4]	45 (61.6%) [48.7–73.3]	28 (38.4%) [26.7–51.3]	0.904
Review	27 (27.0%) [18.6–36.7]	17 (63.0%) [42.4–80.6]	10 (37.0%) [19.4–57.6]	

Temporal distribution showed a concentration of Classics in 2006 (nine publications), coinciding with landmark fMRI and DTI studies on cortical reorganization. Non-Classics contributed more steadily but with lower overall impact.

**Figure 3:**
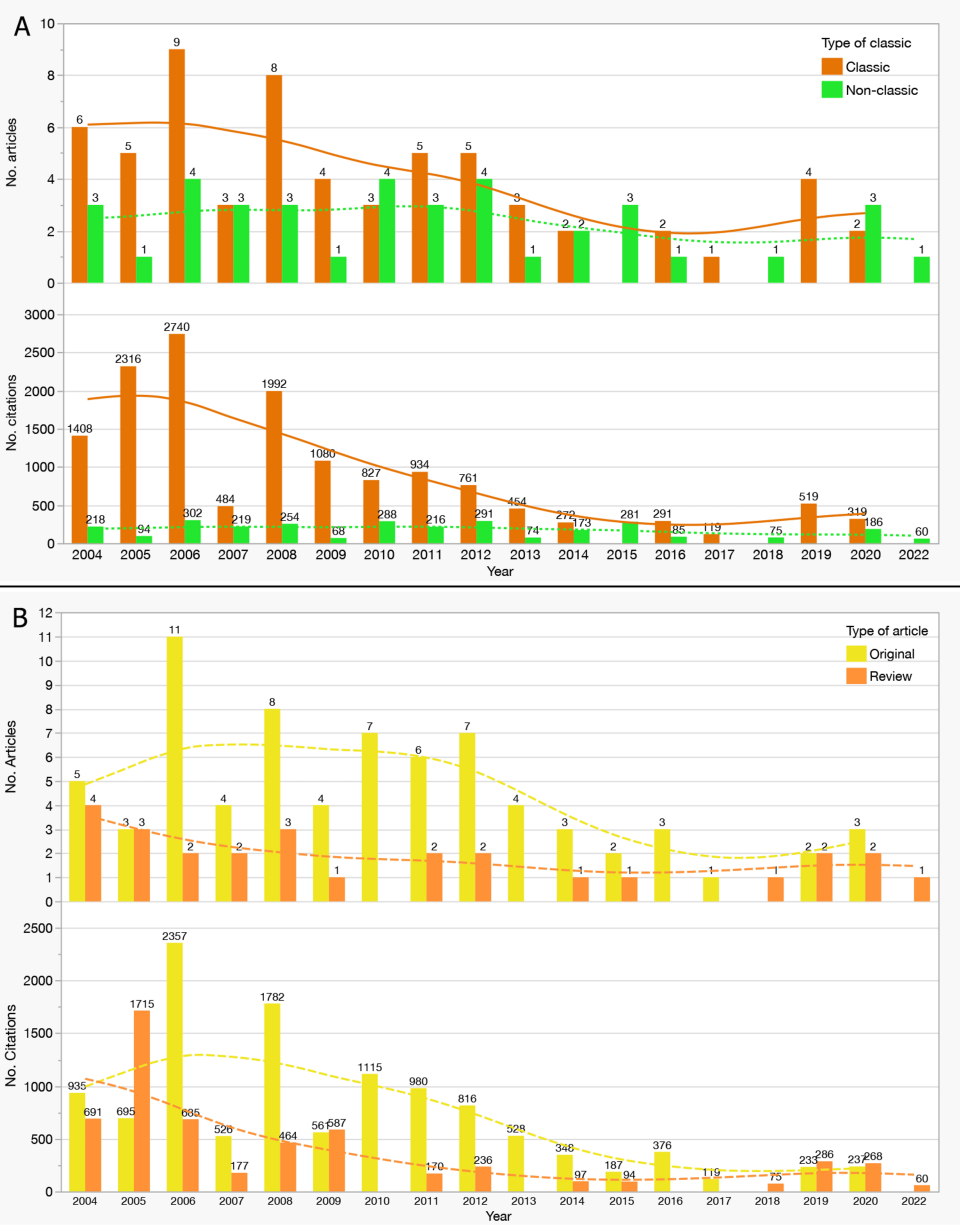
Temporal Distribution of Citation Classics, Non-Classics, Review Articles, and Original Articles among the Top 100 Most-Cited Studies on MRI-Based Neuroplasticity in Stroke (2004–2022). (A) Distribution of Citation Classics (≥100 citations; orange bars) versus Non-Classics (<100 citations; green bars). The upper panel displays the number of articles published per year, while the lower panel shows their annual citation totals. Citation Classics peaked in 2006 with 9 publications and 2,740 citations, followed by 8 publications in 2008. In contrast, Non-Classics maintained a relatively consistent but lower annual output. Dashed trend lines in both panels represent LOESS (Locally Estimated Scatterplot Smoothing) regression curves, illustrating a general decline in both publication frequency and citation volume for Citation Classics over time, with a mild resurgence in 2020. (B) Comparison of Original Articles (yellow bars) and Review Articles (orange bars). The upper panel presents annual publication counts, showing a peak in original articles in 2006 (11 articles) and review articles in 2005 and 2007 (4 articles each). The lower panel depicts annual citation counts, where original articles reached a peak of 2,357 citations in 2006 and sustained higher citation levels through 2012. Review articles exhibited lower overall citation volumes but demonstrated steadier citation rates over time. LOESS regression curves reveal a long-term decline in citations for original research, with a flatter citation trajectory for review articles, suggesting their continued influence in the field. Together, these trends highlight the temporal dynamics of article type and citation performance, underscoring the early dominance of original research in driving citation impact, alongside the persistent relevance of review articles and the waning of hyper-cited classics in recent years.

Figure 3A illustrates the yearly distribution of Citation Classics and Non-Classics in terms of the number of articles. The data reveal a peak in Citation Classics in 2006, with nine publications, marking a period of heightened research activity. In contrast, Non-Classics demonstrated a steady but lower contribution across the years, reflecting their limited impact compared to highly cited works. The trends suggest that seminal publications from earlier years continue to dominate the field.

Figure 3B provides further insights into citation trends, highlighting a pronounced citation peak in 2006, coinciding with the highest number of Citation Classics published. While the citation count for Non-Classics remained consistently low throughout the years, Citation Classics exhibited significant variability, reflecting the disproportionate influence of certain high-impact articles. The declining citation rates for Citation Classics after 2010 may indicate a natural lifecycle of citation accumulation as the field evolves.

Journals and Bibliometric Profiles: Highly cited articles were concentrated in a small group of high-impact journals (Table 5).

**Table 5:** Journals Publishing the Top 100 Most-Cited Articles on MRI-Based Neuroplasticity in Stroke (2004–2024). This table ranks journals that published the most influential articles on MRI applications in stroke-related neuroplasticity research. Data include the total number of articles, distribution by article type (original vs. review), total citation counts, percentage share of citations across the top 100 articles, and average annual citation rate. High-impact neuroscience and neuroimaging journals such as *Brain*, *Stroke*, and *Neurorehabilitation and Neural Repair *dominate the ranking, reflecting their central role in disseminating foundational and translational research. The inclusion of both general neuroscience outlets (e.g., *Neuron*, *Annual Review of Neuroscience*) and specialized clinical journals (e.g., *Neurorehabilitation and Neural Repair*, *Archives of Physical Medicine and Rehabilitation*) highlights the interdisciplinary nature of MRI-based neuroplasticity research in stroke recovery.

Rank	Journal	Articles			Citations		
		Total	Original	Review	Total	%	Average/year
1	Brain	10	10	0	3514	20.2%	20.7
2	Stroke	9	8	1	1744	10.0%	11.1
3	Neurorehabilitation and Neural Repair	9	9	0	1403	8.1%	10.7
4	Annual Review of Neuroscience	2	0	2	1349	7.8%	37.5
5	NeuroImage	7	7	0	982	5.6%	8.6
6	Brain Research Reviews	1	0	1	587	3.4%	37.0
7	Neuropsychologia	4	3	1	450	2.6%	7.0
8	Neuron	2	1	1	408	2.3%	13.0
9	Frontiers in Human Neuroscience	3	3	0	365	2.1%	13.7
10	Physiological Reviews	1	0	1	347	2.0%	17.0
11	Neuroscience	2	1	1	334	1.9%	10.5
12	Stroke; a journal of cerebral circulation	1	1	0	328	1.9%	19.0
13	Cerebral Cortex	3	3	0	325	1.9%	8.7
14	Progress in Neurobiology	1	0	1	325	1.9%	15.0
15	Current Opinion in Neurology	2	0	2	300	1.7%	8.0
16	Neurology	2	2	0	282	1.6%	14.0
17	Journal of Neuroscience	2	2	0	264	1.5%	9.0
18	Frontiers in Neurology	2	0	2	250	1.4%	17.5
19	Restorative Neurology and Neuroscience	3	2	1	227	1.3%	4.3
20	Annals of Neurology	1	1	0	224	1.3%	14.0
21	Europa Medicophysica	2	0	2	213	1.2%	5.5
22	Neurological Research and Practice	1	0	1	209	1.2%	42.0
23	Neurosurgery	1	1	0	197	1.1%	10.0
24	Neural Regeneration Research	2	2	0	193	1.1%	10.5
25	Translational Stroke Research	2	1	1	193	1.1%	14.0
26	Journal of Neurophysiology	1	1	0	184	1.1%	20.0
27	Nature Clinical Practice Neurology	1	0	1	158	0.9%	9.0
28	Psychophysiology	1	1	0	156	0.9%	11.0
29	Cortex	1	1	0	142	0.8%	11.0
30	Physical Medicine and Rehabilitation Clinics of North America	1	0	1	138	0.8%	7.0
31	Clinical Neurophysiology	1	0	1	130	0.7%	22.0
32	Archives of Physical Medicine and Rehabilitation	2	1	1	126	0.7%	11.5
33	Clinical Neurology and Neurosurgery	1	1	0	124	0.7%	10.0
34	NeuroImage: Clinical	1	1	0	116	0.7%	19.0
35	Frontiers in Systems Neuroscience	1	0	1	108	0.6%	8.0
36	Brain Structure and Function	1	1	0	96	0.6%	10.0
37	Neuroimaging Clinics of North America	1	0	1	94	0.5%	5.0
38	Current Neurology and Neuroscience Reports	1	1	0	87	0.5%	5.0
39	Brain Imaging and Behavior	1	1	0	85	0.5%	9.0
40	Journal of Physiology Paris	1	1	0	80	0.5%	4.0
41	Journal of Neurology, Neurosurgery and Psychiatry	1	1	0	68	0.4%	4.0
42	European Journal of Neuroscience	1	1	0	66	0.4%	4.0
43	International Journal of Stroke	1	0	1	66	0.4%	4.0
44	Annals of the New York Academy of Sciences	1	1	0	62	0.4%	5.0
45	NeuroRehabilitation	1	0	1	62	0.4%	4.0
46	Experimental Brain Research	1	1	0	61	0.4%	3.0
47	Seminars in Speech and Language	1	1	0	60	0.3%	4.0
48	Head and Neck	1	1	0	59	0.3%	4.0
49	Neurobiology of Language	1	0	1	59	0.3%	12.0

*Brain *published 10 articles (3,514 citations; 20.2% of total). *Stroke *published nine articles (1,744 citations; 10%). *Neurorehabilitation and Neural Repair* contributed nine articles (1,403 citations; 8.1%). Review journals such as *Annual Review of Neuroscience* and *Brain Research Reviews* also ranked highly, with annual citation rates of 37.5 and 37.0, respectively. Among original research outlets, *NeuroImage *contributed seven studies (982 citations; 5.6%), reflecting its emphasis on advanced MRI techniques.

**Figure 4:**
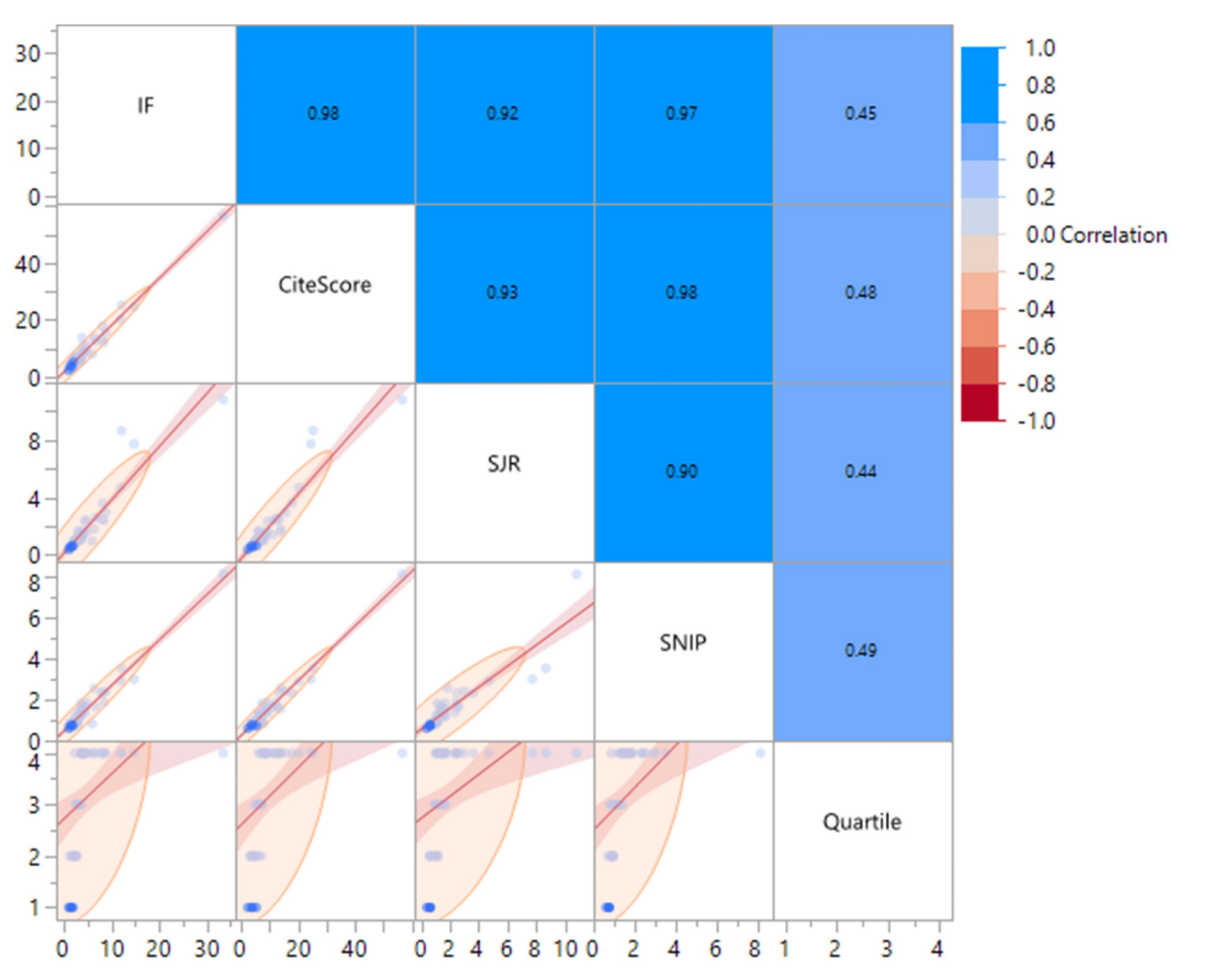
Correlation of Journal Bibliometric Metrics with Citation Performance. This figure illustrates the pairwise Spearman correlation coefficients among five major journal-level bibliometric metrics: Impact Factor (IF), CiteScore, Scimago Journal Rank (SJR), Source Normalized Impact per Paper (SNIP), and Quartile Ranking. The upper triangular matrix shows numerical correlation coefficients, while the lower triangular panels present scatterplots with locally smoothed regression lines. Notably, the strongest observed correlations include IF vs. CiteScore (r = 0.98), IF vs. SNIP (r = 0.97), and CiteScore vs. SNIP (r = 0.98), indicating tight alignment between these metrics in reflecting journal performance. Moderate associations were observed between SJR and SNIP (r = 0.90), and between all metrics and Quartile Rankings (r ≈ 0.44–0.49). These results suggest that the journals contributing most frequently to the highly cited literature in this field tend to occupy top positions across multiple evaluation systems. The high interdependence among these indicators underscores their collective value in assessing journal prestige and influence within the domain of neuroimaging and stroke research.

Correlation analyses demonstrated strong associations between journal bibliometric indices and citation performance: IF and CiteScore (r = 0.98), SNIP (r = 0.97), and SJR (r = 0.92) (Figure 4). 

Most journals belonged to Q1 categories, underscoring the dominance of neuroscience and neurology outlets (Table 6).

**Table 6:** Bibliometric Indicators of Journals Publishing the Top 100 Most-Cited Articles on MRI-Based Neuroplasticity in Stroke. This table summarizes the distribution of journal impact metrics for the sources represented in the study. Reported values include median, 25th percentile, 75th percentile, and interquartile range (IQR) for Impact Factor (IF), CiteScore, SCImago Journal Rank (SJR), and Source Normalized Impact per Paper (SNIP). Quartile classifications (Q1–Q4) are also shown, highlighting that most influential publications were concentrated in Q1 journals. These data reflect the strong representation of high-impact neuroscience and neuroimaging journals in disseminating foundational and translational research on stroke neuroplasticity.

Metric	Median	25th percentile	75th percentile	IQR
Impact Factor (IF)	3.60	2.40	5.63	3.23
CiteScore	7.30	5.53	11.98	6.45
SJR	1.31	0.97	2.19	1.22
SNIP	1.27	0.88	1.78	0.90
Quartile	Q1	Q2	Q4	Q4-Q2

Institutional Contributions: Leading institutions were responsible for a disproportionate share of highly cited work (Table 7).

**Table 7:** Leading Institutions Contributing to MRI-Based Neuroplasticity Research in Stroke. This table ranks the top institutions publishing the most-cited articles on MRI-based neuroplasticity in stroke. For each institution, the number of articles, total citations, percentage of overall citations, and average citations per year are presented. The data highlight the leadership of high-income countries (e.g., Harvard Medical School, University of Cambridge, University of Freiburg, NIH, UC Irvine, King’s College London) while also showing emerging contributions from Asian and Latin American institutions (e.g., Beijing Normal University, Tianjin Medical University, National Autonomous University of Mexico (UNAM)). These findings emphasize both the concentration of research within well-resourced centers and the gradual expansion of global participation.

Rank	Institution	Articles	Citations		
			N	%	Average per year
1	Harvard Medical School	5	1854	10.7%	19.4
2	University of Cambridge	3	877	5.0%	16.7
3	University of Freiburg	1	876	5.0%	46.0
4	National Institute of Neurological Disorders and Stroke, NIH	3	817	4.7%	12.7
5	University of Tübingen	4	785	4.5%	13.5
6	University of California, Irvine	3	733	4.2%	14.0
7	University of California, Los Angeles	4	707	4.1%	9.0
8	University of Giessen	1	587	3.4%	37.0
9	Beijing Normal University	1	527	3.0%	35.0
10	University College London	2	507	2.9%	13.0
11	University of Oxford	3	467	2.7%	12.3
12	University of Alabama	2	430	2.5%	12.0
13	French National Institute of Health and Medical Research	2	422	2.4%	10.5
14	University of South Carolina	3	364	2.1%	8.3
15	King's College London	1	352	2.0%	21.0
16	Hampton University	1	351	2.0%	18.0
17	University of Pennsylvania	2	336	1.9%	12.5
18	University of Cincinnati	3	300	1.7%	6.0
19	Erasmus University Medical Center	1	297	1.7%	21.0
20	Research Centre Jülich	2	277	1.6%	28.0
21	University Medical Center Utrecht	2	271	1.6%	10.0
22	Università Campus Bio-Medico	2	249	1.4%	6.5
23	University of California, San Francisco	2	202	1.2%	6.5
24	Wayne State University	1	197	1.1%	10.0
25	Aalborg University	1	184	1.1%	20.0
26	Northwestern University	2	179	1.0%	5.5
27	University of Minnesota	2	179	1.0%	5.0
28	University of Cologne	2	173	1.0%	8.0
29	Tianjin Medical University	2	167	1.0%	8.0
30	Boston University	1	156	0.9%	26.0
31	University of Florida	1	156	0.9%	9.0
32	The Rockefeller University	1	153	0.9%	12.0
33	Université de Bordeaux	1	152	0.9%	7.0
34	University of Maryland	1	150	0.9%	11.0
35	Georgetown University	1	142	0.8%	11.0
36	University Medical Center Hamburg Eppendorf	1	138	0.8%	7.0
37	Institut Universitaire de Gériatrie de Montréal	2	136	0.8%	5.0
38	University of Helsinki	1	135	0.8%	12.0
39	University Hospital Geneva	1	130	0.7%	22.0
40	University Hospital RWTH Aachen	1	126	0.7%	7.0
41	All India Institute of Medical Sciences	1	124	0.7%	10.0
42	University Clinic of Jena	1	122	0.7%	9.0
43	Capital Medical University	1	119	0.7%	15.0
44	University of Southern California	1	117	0.7%	20.0
45	Nanjing University	1	116	0.7%	19.0
46	Université Grenoble Alpes	1	110	0.6%	22.0
47	Henry Ford Hospital	1	105	0.6%	7.0
48	Universidad Nacional Autónoma de México	1	97	0.6%	9.0
49	Max Planck Institute for Neurological Research	1	96	0.6%	10.0
50	University of British Columbia	1	94	0.5%	9.0
51	Medical University of Graz	1	92	0.5%	5.0
52	University of Barcelona	1	85	0.5%	9.0
53	University Hospital of Düsseldorf	1	80	0.5%	4.0
54	University Medical Center Hamburg-Eppendorf	1	79	0.5%	4.0
55	Essen University Hospital	1	78	0.4%	4.0
56	Massachusetts Institute of Technology	1	76	0.4%	4.0
57	Sun Yat-sen University	1	74	0.4%	6.0
58	Sichuan University	1	68	0.4%	4.0
59	Rehabilitation Institute of Chicago	1	66	0.4%	4.0
60	Beth Israel Deaconess Medical Center	1	62	0.4%	5.0
61	Yeungnam University	1	62	0.4%	4.0
62	University of Nebraska Medical Center	1	60	0.3%	20.0
63	Columbia University	1	59	0.3%	4.0
64	Fudan University	1	59	0.3%	12.0
65	Vanderbilt University Medical Center	1	59	0.3%	12.0

Harvard Medical School produced five articles (1,854 citations; 10.7%). The University of Cambridge contributed three articles (877 citations). University of Freiburg achieved 876 citations with a single paper, yielding the highest per-year average (46.0). Prominent U.S. centers included the National Institute of Neurological Disorders and Stroke (NINDS) (817 citations) and UC Irvine (733 citations). European leaders included Tübingen, UCL, and Oxford. Beijing Normal University represented emerging Asian participation with one article (527 citations; 35/year).

These findings reflect both resource concentration in high-income countries and growing engagement from non-Western institutions.

**Figure 5:**
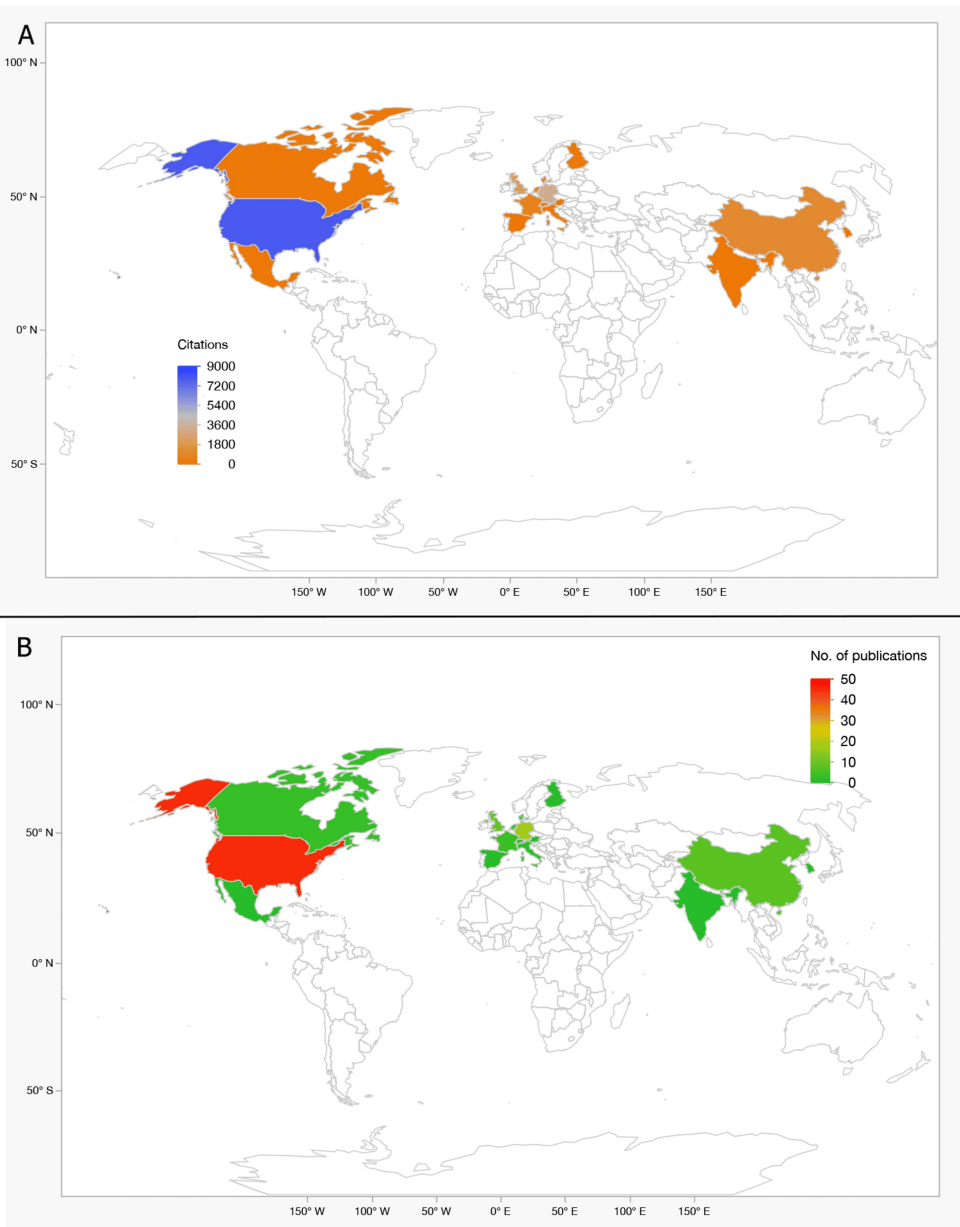
Geographic Distribution of Publications and Citations (2004–2024). (A) Global citation map showing the United States as the leading contributor (46% of total citations, n = 8,010). (B) Publications by country, with Germany (17 articles, 3,417 citations) and the United Kingdom (9 articles, 2,203 citations) as major contributors. Emerging outputs from China and Japan highlight growing international engagement.

Country-Level Contributions: Geographic analysis confirmed the dominance of the United States, contributing 46 articles and 8,010 citations (46%). Germany ranked second with 17 articles (3,417 citations; 19.6%), followed by the UK with nine articles (2,203 citations; 12.7%) (Table 8, Figure 5).

**Table 8:** Geographic Distribution of Top-Cited Articles in MRI-Based Neuroplasticity Research in Stroke. This table summarizes the country-level contributions to the top 100 most-cited articles, reporting the number of publications, total citations, percentage of global citations, and average citations per year. The United States led with 46 articles and 46% of all citations, followed by Germany, the United Kingdom, and China. European countries such as France and the Netherlands also had strong citation performance, while emerging contributors—including China, India, Mexico, and South Korea—reflect growing international participation. High average yearly citation rates in smaller contributors such as Denmark and Switzerland highlight the impact of select influential studies.

Ranking	Country	Articles		Citations		
			Number		%	Average per year
1	United States	46	8010		46.0%	11.1
2	Germany	17	3417		19.6%	14.9
3	United Kingdom	9	2203		12.7%	14.9
4	China	8	1130		6.5%	13.4
5	France	4	684		3.9%	12.5
6	Netherlands	3	568		3.3%	13.7
7	Italy	2	249		1.4%	6.5
8	Canada	3	230		1.3%	6.3
9	Denmark	1	184		1.1%	20.0
10	Finland	1	135		0.8%	12.0
11	Switzerland	1	130		0.7%	22.0
12	India	1	124		0.7%	10.0
13	Mexico	1	97		0.6%	9.0
14	Austria	1	92		0.5%	5.0
15	Spain	1	85		0.5%	9.0
16	South Korea	1	62		0.4%	4.0

China ranked fourth with eight publications (1,130 citations; 6.5%), reflecting rapid expansion in neuroimaging research. Other notable contributors included France, the Netherlands, and Canada.

Smaller nations made high-impact contributions: Denmark (20 citations/year) and Switzerland (22/year) produced single but influential papers. Emerging economies such as India, Mexico, and South Korea showed modest output but promising average citations (~9-10 per year).

Cross-country bibliographic coupling indicated strong collaborations among the U.S., UK, and Germany, but limited representation from Latin America and Africa.

Science Mapping

**Figure 6:**
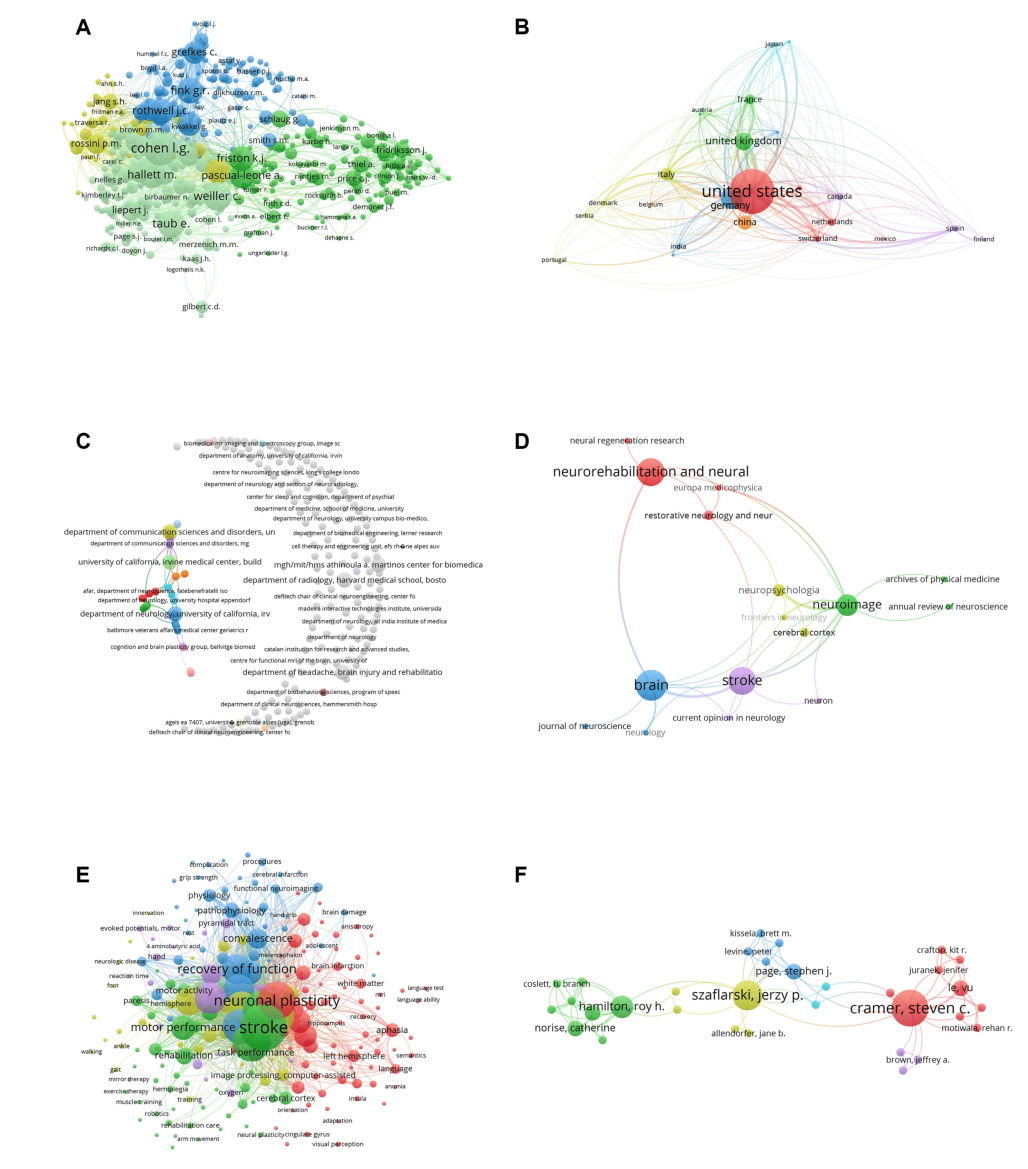
Science Mapping of MRI-Based Neuroplasticity Research. (A) Author co-citation network (207 authors, ≥5 co-citations) clustering around Cohen L.G., Friston K.J., and Pascual-Leone A. (B) Country-level co-authorship network (25 countries), with the U.S., Germany, and the U.K. as central hubs. (C) Institutional co-authorship network (120 institutions), highlighting Harvard Medical School, University of California, and University of Freiburg. (D) Source journal co-citation map showing five clusters; key sources include *Brain*, *Stroke*, and *Neurorehabilitation and Neural Repair*. (E) Keyword co-occurrence map (112 terms, ≥5 occurrences) identifying clusters on motor recovery, cortical reorganization, language/aphasia, and neurophysiology. (F) Author-level co-authorship network (56 authors), led by Cramer, Szaflarski, and Hamilton, emphasizing collaborations in motor rehabilitation and cortical reorganization. Visualization notes: Node size = frequency (occurrences or citations); node color = cluster membership from co-occurrence or co-citation analysis; line thickness = strength of the relationship (e.g., collaboration intensity, citation link, or keyword co-appearance). Collectively, these features depict the structural and intellectual landscape of the field.

Intellectual Structure of the Field: The intellectual structure of MRI-based research on stroke neuroplasticity was visualized through co-citation and bibliographic coupling networks (Figure 6A-6D).

Author co-citation network (Figure 6A) - Central nodes included Cohen L.G., Pascual-Leone A., and Friston K.J., whose foundational contributions span brain plasticity, neurorehabilitation, and advanced modeling. Other frequently co-cited authors such as Rothwell J.C., Rossini P.M., and Taub E. formed interconnected clusters emphasizing cortical stimulation, fMRI-based recovery, and rehabilitation theory. Authors including Grefkes C., Hallett M., and Weiller C. highlighted integration of MRI with clinical neurorehabilitation.

Country-level bibliographic coupling (Figure 6B) - The United States dominated output and collaborations, followed by Germany, the UK, and China. Strong cross-links also involved France, Italy, and Canada. Emerging clusters in Japan, Spain, and India underscored rising international participation.

Institutional network (Figure 6C) - Harvard Medical School and UC Irvine emerged as hubs, with additional key contributions from King’s College London and the Massachusetts General Hospital/Massachusetts Institute of Technology (MGH/MIT) Athinoula A. Martinos Center. These institutions exemplify the importance of multidisciplinary environments combining neurology, radiology, and neuroscience.

Journal co-citation map (Figure 6D) - Core journals included *Brain*, *Stroke*, *NeuroImage*, and *Neurorehabilitation and Neural Repair*. Strong co-citation ties were observed between *Brain *and *Stroke*, and between *NeuroImage *and *Neuropsychologia*. Additional influential outlets included *Neuron*, *Cerebral Cortex*, *Annual Review of Neuroscience*, and *Neurology*.

Conceptual Structure and Research Themes: Keyword co-occurrence analysis revealed distinct thematic clusters (Figure 6E):

Motor recovery and rehabilitation - Central terms included “neuroplasticity,” “stroke,” “motor performance,” and “recovery of function,” linked with “gait rehabilitation,” “mirror therapy,” “robotics,” and “muscle training.”

Cortical reorganization and imaging - Anchored by “cerebral cortex,” “functional neuroimaging,” and “image processing,” this cluster linked imaging findings with outcomes such as pyramidal tract integrity, oxygenation, and innervation.

Language and aphasia - Terms included “aphasia,” “semantics,” “white matter,” and “hippocampus,” with related concepts such as “anomia” and “language testing.”

Neurophysiology and recovery mechanisms - A smaller cluster that captured concepts such as “motor evoked potentials,” “reaction time,” and “grip strength,” reflecting integration of neuroimaging with physiological measures.

These clusters collectively illustrate the integration of structural, functional, and behavioral dimensions in MRI-based stroke neuroplasticity research.

Collaboration Networks: Author-level co-authorship networks (Figure 6F) highlighted the collaborative nature of the field.

The largest cluster was led by Steven C. Cramer, with extensive collaborations on imaging-based stroke recovery.

Jerzy P. Szaflarski represented another hub, bridging groups focused on motor recovery, functional connectivity, and cognitive outcomes.

A smaller but cohesive cluster led by Roy H. Hamilton and Catherine Norise emphasized non-invasive brain stimulation and cognitive rehabilitation, supported by contributions from Coslett H. Branch.

Another subgroup led by Page Stephen J. and Kissela Brett M. focused on motor recovery and neuroimaging, connecting closely with Szaflarski’s cluster.

Overall, the collaboration landscape displayed a hierarchical yet interconnected structure, with Cramer, Szaflarski, and Hamilton serving as pivotal figures. Smaller clusters complemented these hubs, reflecting both breadth and specialization across institutions and research foci.

Discussion

Overview of Performance Trends

The retrieval and selection of the top 100 most-cited articles underscore the pivotal role of MRI in advancing the understanding of neuroplasticity after stroke. This bibliometric focus highlights not only methodological rigor but also the intellectual and clinical influence of MRI-based investigations over the last two decades. Identifying highly cited works provides a framework for recognizing research trends, leading authors, and key institutions, while also guiding future studies toward emerging imaging modalities and multidisciplinary approaches in neurorehabilitation.

Publication and Citation Patterns

Annual publication and citation trends revealed distinct phases in the evolution of MRI-based neuroplasticity research. The surge in 2006 reflected a turning point marked by fMRI mapping of motor recovery and DTI assessment of white matter integrity [33,62,65]. These seminal contributions established MRI as a cornerstone of stroke rehabilitation research. Between 2004 and 2012, steady productivity sustained advances in cortical reorganization and recovery mechanisms [36,38,51]. After 2012, declines in both publications and citations suggested a shift toward newer imaging techniques and rehabilitation strategies, though landmark works from the mid-2000s continued to dominate. Citation rebounds in 2019-2020 underscored the enduring impact of studies on functional connectivity and advanced MRI-based monitoring of neuroplastic changes [2,75,78,124]. These dynamics confirm the lasting influence of early contributions while highlighting opportunities for innovation through connectomics, multi-modal imaging, and longitudinal approaches.

Citation Classics vs. Non-Classics

Comparisons between Citation Classics (≥100 citations) and non-Classics showed that Classics achieved higher citation rates per year, underscoring their sustained relevance. Reviews were more common among Classics, synthesizing evidence on fMRI, DTI, and resting-state functional connectivity [29,35,37]. In contrast, Citation Classics often represented original studies showcasing detailed clinical or technical applications [45,63,66,71]. The peak period of Classics around 2006 corresponded to landmark studies on perilesional cortical recruitment and hemispheric activation shifts [70,100,112]. Their subsequent decline likely reflects thematic saturation and the difficulty of producing equally influential work. Future high-impact contributions may arise from combining MRI with complementary modalities such as EEG and MEG, integrating neuromodulation approaches like TMS and tDCS, and applying machine learning to predictive rehabilitation models.

Journals and Dissemination Platforms

The analysis reinforced the dominance of a small number of high-impact journals. *NeuroImage*, *Brain*, and *Stroke *accounted for the majority of influential publications, particularly those advancing fMRI and DTI [14,46,48]. Specialized outlets, including *Neurorehabilitation and Neural Repair *and *Archives of Physical Medicine and Rehabilitation*, emphasized translational studies on therapies, telerehabilitation, and training interventions [60,72,112]. Correlations between journal bibliometric metrics and citation performance highlighted the visibility advantage of top-ranked journals. While broad-scope journals ensured wide dissemination, selective outlets such as *Cerebral Cortex *and *Restorative Neurology and Neuroscience *maintained strong citation averages despite smaller publication volumes. Highly cited works addressed cortical reorganization, functional recovery, and structural integrity [34,71,85], while more recent themes - connectomics, multimodal imaging, and network plasticity - signal evolving directions for impactful dissemination [56,81,107]. 

Institutional and Geographic Contributions

Institutions such as UC Irvine, Harvard Medical School, and King’s College London consistently drove highly cited research, supported by infrastructure and interdisciplinary collaborations. UC Irvine contributed extensively to fMRI and DTI studies of cortical reorganization, while Harvard advanced resting-state connectivity research through the MGH/MIT Athinoula A. Martinos Center. King’s College London focused on white matter integrity and structural plasticity. European centers such as Oxford and Tübingen added expertise in cognitive recovery and non-invasive stimulation.

At the national level, the United States dominated both output and citations, followed by the UK and Germany. China, Japan, and Canada showed increasing productivity, with Chinese teams integrating multimodal MRI and machine learning, and Japanese groups emphasizing cerebral reorganization and rehabilitation outcomes. Strong bibliographic coupling revealed extensive collaborations among the US, UK, and Germany, but limited participation of Latin America and Africa. Expanding collaborative networks in these regions remains essential to reducing disparities and ensuring global applicability of findings.

Science Mapping and Intellectual Structure

Science mapping revealed an interconnected research landscape shaped by leading institutions and authors. Harvard, UC Irvine, and King’s College London emerged as intellectual hubs, advancing fMRI, resting-state connectivity, and white matter integrity studies. Influential figures such as Pascual-Leone, with seminal work on non-invasive brain stimulation [125-128], and Friston, through dynamic causal modeling [129-132], established frameworks that integrated neuroimaging with neurophysiological insights. Core journals, including *Brain*, *Stroke*, and *NeuroImage*,* *served as key platforms consolidating progress and visibility.

Conceptual Clusters and Emerging Areas

Keyword mapping highlighted clusters centered on neuroplasticity, motor recovery, and structural connectivity, reaffirming the importance of fMRI and DTI in stroke rehabilitation. Emerging themes included connectomics and neurochemical imaging with MRS, reflecting a transition toward large-scale brain network mapping and biochemical profiling [2,89]. Translational priorities were evident in clusters on language recovery, robotic rehabilitation, and task-based neuroplasticity [45,49,76,101,122], underscoring the effort to bridge imaging insights with clinical outcomes.

Collaboration Networks

Collaboration patterns showed highly interconnected structures. Steven C. Cramer emerged as a central figure in motor recovery and imaging-based rehabilitation [61,133], while Jerzy Szaflarski bridged domains of functional connectivity and cognitive recovery [112]. Roy H. Hamilton and collaborators contributed significantly to non-invasive brain stimulation [134]. Strong cross-national collaborations were evident among the US, UK, and Germany, though Latin America and Africa remained underrepresented. Multinational teams increasingly addressed novel technologies such as brain-computer interfaces, virtual reality, and integrative rehabilitation strategies [42,49,106,110].

International collaborations were strongly associated with higher citation impact, reflecting the synergistic benefits of shared expertise, access to advanced imaging infrastructure, and broader dissemination across multiple networks. However, the underrepresentation of Asia, Africa, and South America likely reflects both resource disparities in neuroimaging availability and database indexing biases that favor North American and European outputs. Strengthening global partnerships and expanding indexing inclusivity will be essential for broadening the scope and clinical applicability of MRI-based neuroplasticity research.

Evolution of Neuroplasticity Research

Between 2010-2015, research focused on motor recovery and cortical reorganization, establishing neuroplasticity as a foundation of rehabilitation science. From 2016-2020, multimodal imaging and brain stimulation gained prominence, reflecting interest in combining MRI with neuromodulatory and neurophysiological methods. Since 2021, connectomics and machine learning have risen rapidly, signaling a paradigm shift toward computational neuroscience and predictive modeling.

**Figure 7:**
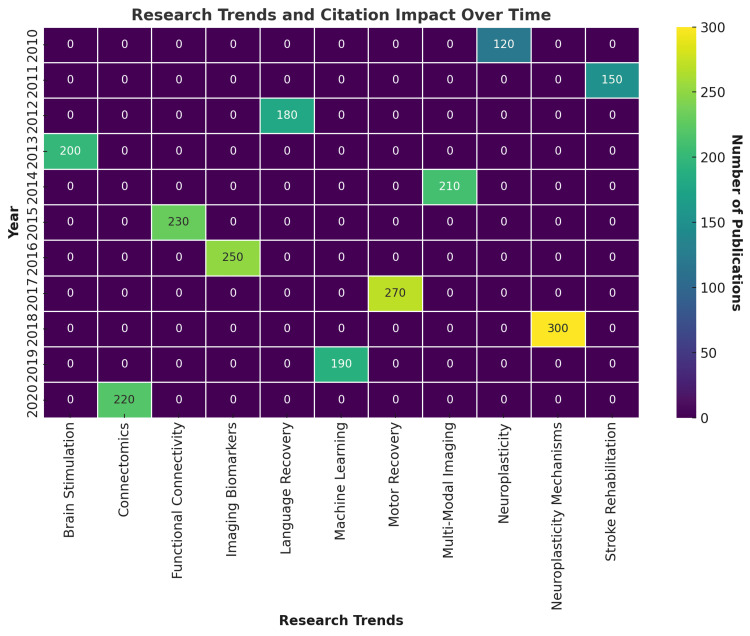
Evolution of Research Trends and Citation Impact. This heatmap illustrates the annual distribution of citation-weighted publications across ten major research domains in neuroplasticity from 2010 to 2020. The x-axis categorizes research trends—Brain Stimulation, Connectomics, Functional Connectivity, Imaging Biomarkers, Language Recovery, Machine Learning, Motor Recovery, Multi-Modal Imaging, Neuroplasticity Mechanisms, and Stroke Rehabilitation—while the y-axis denotes publication year. Color intensity reflects the number of articles and their associated citation impact, with warmer tones (yellow) indicating higher activity. Thematic analysis reveals a notable rise in Connectomics and Machine Learning after 2019, suggesting a growing integration of computational modeling and network science into neuroplasticity research. Motor Recovery exhibited consistent representation, peaking in 2020 with 300 citations, while Imaging Biomarkers showed a sharp increase in 2016 (250 citations). Established topics such as Neuroplasticity Mechanisms and Brain Stimulation maintained intermittent yet impactful contributions throughout the decade. This visualization underscores evolving research priorities and the shifting focus toward interdisciplinary, data-driven approaches in stroke recovery and neurorehabilitation.

Citation trajectories (Figure 7) showed successive waves of influence: early dominance of functional connectivity, followed by multimodal integration, and most recently, computational approaches. Connectomics and machine learning studies have achieved the highest recent citation rates, while functional connectivity and imaging biomarkers remain enduring anchors.

Recent Advances and Translational Innovations

Recent progress includes improved diagnostic tools, collaborative research, and therapeutic innovation. High-resolution fMRI and PET now enable earlier identification of dysfunctional networks [135], while machine learning augments prognostic modeling [136]. International collaborations have facilitated VR-based telerehabilitation [137], expanding access and engagement. Therapeutic advances include non-invasive brain stimulation (TMS, tDCS), robotic-assisted therapy, and immersive VR platforms [138-140], each enhancing recovery and real-world skill transfer. These advances demonstrate how neuroplasticity research continues to shape clinical practice.

Limitations

This study has several limitations. Reliance on citation counts privileges older, highly cited works and may underrepresent recent but high-quality contributions. Although citation rates per year were reported to mitigate age-related bias, field-weighted metrics were not applied. The top-100 cutoff may also exclude niche but relevant studies, such as those on connectomics or longitudinal designs. Exclusive reliance on Scopus and restriction to English-language publications introduce database and language bias, while the absence of dual independent screening and reliance on a single primary reviewer raise the possibility of selection bias. Finally, the dominance of high-income countries highlights disparities in global access to imaging, and the study did not evaluate methodological rigor or clinical applicability, limiting interpretability for direct patient outcomes.

Future directions

The intellectual and collaborative structures in this field reflect strong foundations and promising new directions. Advances in connectomics and multimodal imaging offer opportunities to uncover novel biomarkers, while qualitative assessments of study design and translational outcomes will improve clinical relevance. Expanding global collaboration, especially with underrepresented regions, remains critical for equitable progress. By combining historical insights with emerging computational tools, MRI-based neuroplasticity research is well-positioned to drive innovation in personalized rehabilitation and long-term recovery after stroke.

## Conclusions

This bibliometric analysis offers a comprehensive overview of the intellectual landscape, key concepts, and collaborative networks that have shaped MRI-based neuroplasticity research in stroke. By examining the 100 most-cited articles, the study highlights the central role of fMRI and DTI in elucidating cortical reorganization, structural connectivity, and motor recovery mechanisms. Contributions from leading institutions such as Harvard Medical School and the University of California, Irvine underscore the importance of interdisciplinary collaboration and methodological innovation in advancing the field.

The findings also reveal the concentration of influential research in high-impact journals, including *Brain*, *Stroke*, and *NeuroImage*,* *which serve as primary platforms for disseminating both foundational and translational advances. Prominent authors such as Steven C. Cramer and Jerzy Szaflarski exemplify the collaborative networks that have accelerated clinical translation and sustained academic influence.

Although overall publication and citation activity have declined in recent years, the rapid growth of connectomics, multimodal imaging, and machine learning approaches signals a promising revitalization of the field. Expanding international collaborations, particularly with underrepresented regions, will be essential for ensuring inclusivity and global applicability of findings.

In conclusion, MRI continues to serve as a cornerstone in stroke neuroplasticity research, bridging mechanistic insights with clinical applications. Future progress will depend on integrating advanced imaging modalities, fostering multidisciplinary networks, and prioritizing translational outcomes to enhance rehabilitation strategies and ultimately improve patient care.
